# Complete and safe resection of challenging retroperitoneal tumors: anticipation of multi-organ and major vascular resection and use of adjunct procedures

**DOI:** 10.1186/1477-7819-9-143

**Published:** 2011-11-04

**Authors:** William W Tseng, Sam C Wang, Charles M Eichler, Robert S Warren, Eric K Nakakura

**Affiliations:** 1 Divisions of Surgical Oncology, University of California at San Francisco (UCSF), 505 Parnassus Avenue, San Francisco, CA 94143 USA; 2 Vascular Surgery, Department of Surgery, University of California at San Francisco (UCSF), 505 Parnassus Avenue, San Francisco, CA 94143 USA; 3 Current address: Department of Surgical Oncology, University of Texas MD Anderson Cancer Center, 1515 Holcombe Blvd, Houston, Texas 77030 USA

**Keywords:** Retroperitoneal Tumor, Multi-organ Resection, Adjunct Procedures

## Abstract

**Background:**

Retroperitoneal tumors are often massive and can involve adjacent organs and/or vital structures, making them difficult to resect. Completeness of resection is within the surgeon's control and critical for long-term survival, particularly for malignant disease. Few studies directly address strategies for complete and safe resection of challenging retroperitoneal tumors.

**Methods:**

Fifty-six patients representing 63 cases of primary or recurrent retroperitoneal tumor resection between 2004-2009 were identified and a retrospective chart review was performed. Rates of complete resection, use of adjunct procedures, and perioperative complications were recorded.

**Results:**

In 95% of cases, complete resection was achieved. Fifty-eight percent of these cases required en bloc multi-organ resection, and 8% required major vascular resection. Complete resection rates were higher for primary versus recurrent disease. Adjunct procedures (ureteral stents, femoral nerve monitoring, posterior laminotomy, etc.) were used in 54% of cases. Major postoperative complications occurred in 16% of cases, and one patient died (2% mortality).

**Conclusions:**

Complete resection of challenging retroperitoneal tumors is feasible and can be done safely with important pre- and intraoperative considerations in mind.

## Background

Retroperitoneal tumors are relatively uncommon but can be very challenging to manage, even for the experienced surgeon. For malignant disease, which accounts for most retroperitoneal tumors [1,2], prognosis depends on tumor grade and histologic subtype, completeness of resection, and presence of distant metastases [3-6]. These factors are for the most part, dependent on tumor biology; however, the ability to completely resect a retroperitoneal tumor is within the control of the surgeon and has tremendous impact on long-term survival [3,4,6]. With greater experience and improved operative technique, many centers have recently reported complete resection rates over 90% and, consequently, better clinical outcome [7-9]. However, as retroperitoneal tumors can be enormous and often involve multiple adjacent organs and vital structures, successful complete resection must also be carefully balanced with patient safety.

Few studies directly address strategies for complete and safe resection of challenging retroperitoneal tumors. In this retrospective review, we describe important pre- and intraoperative considerations based on our recent experience with challenging retroperitoneal tumors in patients at a tertiary referral center.

## Materials and methods

After obtaining institutional review board approval, we identified 56 adult patients with primary or recurrent retroperitoneal tumors who underwent a total of 63 resections at the University of California San Francisco Medical Center between 2004 and 2009. Their charts were retrospectively reviewed to determine rates of complete resection and perioperative complications. Specific attention was also given to adjunct procedures used to improve resectability and minimize complications. Data was also collected regarding patient demographics (age, sex), tumor characteristics (size, pathology, grade), operative details (blood loss, operative time) and hospital course (length of stay).

Before surgery, all patients underwent a thorough evaluation of co-morbid conditions, and cardiac and pulmonary function were optimized. Contrast-enhanced computed tomographic scanning or magnetic resonance imaging was done to evaluate the local extent of tumor and rule out presence of metastatic disease before resection. On the basis of their preoperative imaging results, patients were further evaluated by consulting services as needed (e.g., urology for ureteral stent placement and/or nephrectomy; vascular surgery for major vessel resection, graft selection/sizing and reconstruction; neurosurgery for potential spinal nerve root involvement). Preoperative tissue diagnosis was available in some patients but was not required before surgery if the result would not have affected patient care.

## Results

Median age for all patients was 52 years (range 22-77) with an even distribution of male and female patients. Out of 63 retroperitoneal tumor resections, 35 (56%) were performed for primary disease, 28 (44%) for recurrence. Median operative time was 316 minutes (range 149-759). Median estimated blood loss was 500 cc (range 50-25,000).

**Figure 1:**
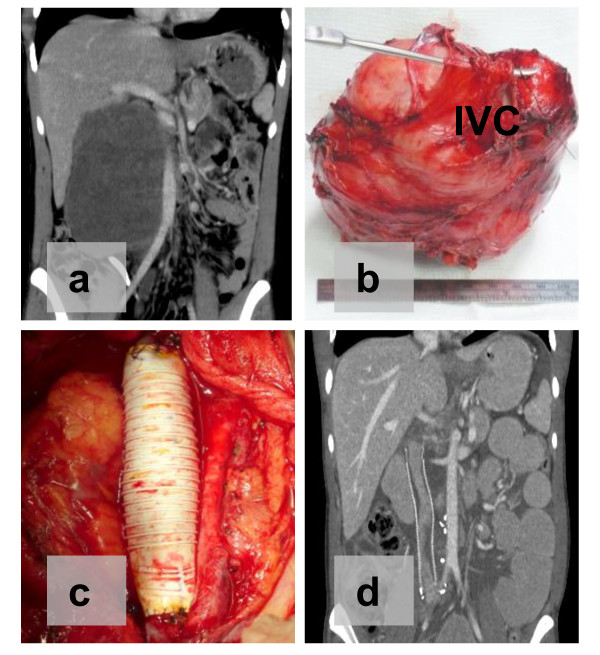
**Retroperitoneal Tumor Involvement of the Inferior Vena Cava (IVC)**. Preoperative computed tomography scan demonstrates IVC involvement without obvious venous collateralization (a). The tumor was resected en bloc with a portion of the IVC (b) and reconstructed using synthetic polytetrafluoroethylene (PTFE) tube graft (c). Follow-up imaging was done postoperatively (d).

Complete resection, as defined by macroscopically negative margins (R0-R1), was achieved in 95% of all operations performed (Table 1). In three patients, complete resection was not possible because of extensive peritoneal sarcomatosis at laparotomy (n = 2) and significant intraoperative bleeding (n = 1, discussed below). Complete resection was feasible, but achieved slightly less often in resections performed for recurrent tumors (93%) versus primary tumors (97%, Table 1). Tumor involvement of adjacent organs necessitated concomitant en bloc resection to achieve macroscopically negative margins in 58% of all cases (Table 1). The kidney, colon, and pancreas were the most frequently involved and resected organs. In 8% of cases, major vascular (e.g. inferior vena cava, iliac vessel) resection was required. In each case, anatomic vascular reconstruction was done immediately after resection by using synthetic tube grafts (for inferior vena cava) or cryopreserved human vein (for iliac vessels) (Figure 1). Among patients who had complete resection, the rates of multi-organ or major vascular resection were higher in those with primary disease (71%; n = 24/34) than in those with recurrent disease (62%; n = 16/26).

**Table 1:** Operative Details

	No. of cases (%)		
**Complete Resection**	**Total**	**Primary**	**Recurrent**
**(out of n = 63 cases: 35 primary, 28 recurrent)**	****60 (95)****	**34 (97)**	**26 (93)**

**Multi-organ Resection**	**35 (58)**	21 (62)	14 (54)
(out of n = 60 cases with complete resection)			
Kidney	18 (30)	10	8
Colon	10 (17)	3	7
Pancreas	4 (7)	3	1
Small bowel	3 (5)	1	2
Adrenal	3 (5)	3	0
Bladder	2 (3)	2	0
Liver	1 (2)	1	0
Spleen	1 (2)	1	0
Diaphragm	1 (2)	1	0
Iliac wing	1 (2)	0	1
**Major Vascular resection**	**5 (8)**	3 (9)	2 (8)
(out of n = 60 cases with complete resection)			
IVC	4 (7)	3	1
Iliac artery, vein	1 (2)	0	1

**Adjunct Procedures**			
(out of n = 63 cases)			
Posterior laminotomy	3 (5)	2	1
Angioembolization	2 (3)	2	0
Ureteral stent	22 (35)	14	8
Femoral nerve monitoring	7 (11)	3	4

Several pre-operative and intraoperative adjunct procedures were used to facilitate safe and complete resection in 54% of cases (Table 1). The decision to use any of these procedures was based on careful review of preoperative imaging studies that raised concern for tumor involvement of critical structures. For example, in three patients, a posterior laminotomy was done by neurosurgeons because preoperative imaging suggested spinal nerve root involvement. In two patients whose CT scans suggested high tumor vascularity, preoperative angioembolization was done by interventional radiologists to limit intraoperative blood loss. To help identify and avoid iatrogenic injury to the ureter, ureteral stents were placed by urologists before laparotomy in 35% of resections. Intraoperative femoral nerve monitoring, which we described previously [10], was done in 11% of cases to help identify the femoral nerve and prevent postoperative disability when involvement was suspected based on the location of the tumor and/or symptoms (e.g. paresthesias, pain, weakness).

With the exception of one patient with significant intraoperative bleeding (discussed below), all patients tolerated resection of their retroperitoneal tumor without intraoperative complications. A total of 24 complications were observed in the postoperative period. When scored according to the revised Accordion Classification scheme [11], the majority of complications were mild to moderate (Grade 1-3, Table 2). Major postoperative complications, as defined by organ failure, need for reoperation, or death (Grade 4-6), occurred in 16% of cases. The most common complication overall was intra-abdominal abscess, which was managed by percutaneous drainage and parenteral antibiotics in all cases. The second most common complication was postoperative atrial fibrillation, which occurred in 4 patients. One patient had pre-existing atrial fibrillation, and none experienced a myocardial infarction. Atrial fibrillation was thought to be related to perioperative intravascular volume shifts and atrial stretch.

**Table 2:** Complications

**Revised Accordion Classification [ref **[10]]		
Grade	Type/Intervention Needed	No. of cases
1	Minor procedures done at bedside	2
2	Pharmacologic treatment, blood transfusions, TPN	12
3	Endoscopic or interventional procedure	6
4	General anesthesia, single organ failure	3
5	Multi-organ (>2) organ failure	0
6	Death	1
	Total	**24**

One patient had significant intraoperative bleeding that precluded complete resection and ultimately died (2% 30-day series mortality). This patient had a primary epithelioid tumor with invasion into the vagina and rectum that required partial vaginectomy and abdominoperineal resection. Tumor dissection close to the sacrum led to profuse venous bleeding and massive blood transfusion requirements that necessitated damage control measures and cessation of surgery. The patient developed multi-organ failure and died on postoperative day four.

In total, median length of hospital stay for all patients was 7 days (range 4-72). 38% (24/63) required postoperative recovery in the intensive care unit and among those patients, median length of stay was 4 days (range 1-23).

**Figure 2:**
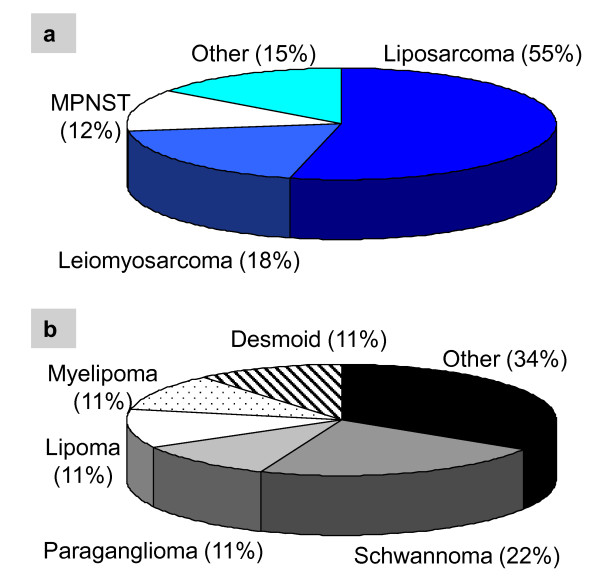
**Final Pathology of Retroperitoneal Tumors**. Among malignant tumors (a), sarcoma was the most common diagnosis. A variety of pathology was encountered for benign tumors (b). MPNST = malignant peripheral nerve sheath tumor.

The final pathology results of resected retroperitoneal tumors revealed that 69% of cases had malignant disease. Among the malignant tumors, retroperitoneal sarcoma was the most common diagnosis (72%, n = 32/44). Liposarcoma was the most common sarcoma subtype (55%; n = 24/44), followed by leiomyosarcoma (18%; n = 8/44) (Figure 2a). Rare diagnoses such as undifferentiated pleomorphic sarcomas and spindle cell sarcomas were also seen. Among the benign tumors, schwannomas and paragangliomas were the most frequent (Figure 2b). Tumor sizes ranged from 2 to 48 cm, with a median of 14 cm. Only four tumors (7%) were less than 5 cm.

## Discussion

Retroperitoneal tumors can be very challenging to manage. Large, single institution case series of retroperitoneal tumors suggest that most are malignant [1,2]. For malignant disease (i.e., sarcomas), completeness of resection is a critical prognostic factor for survival [3,4,6]. In fact, a recently proposed revised staging system and a survival nomogram both incorporate completeness of resection [12,13]. However, because retroperitoneal tumors are often enormous and close to critical retroperitoneal structures and organs, complete resection is difficult and the potential for perioperative complications is high. The results of our study suggest that for most patients, complete and safe resection is possible, but multi-organ and major vascular resections are frequently required and several adjunct procedures may be utilized to minimize complications.

For retroperitoneal tumors, large case series indicate that multi-organ resection is to be anticipated in up to 80% of patients, with the kidney being the most common organ resected en bloc with the primary tumor [3,4,6]. Although preoperative imaging studies may suggest adjacent organ involvement, often, definitive assessment can only be made intraoperatively. This underscores the importance of preoperative planning, especially if the visceral organs to be resected are outside of the surgeon's area of technical expertise. Assistance from consulting services may be needed.

Recently, Bonvalot et al. have advocated "complete compartmental surgery", which involves multi-organ resection, even without obvious tumor involvement of adjacent organs at the time of surgical exploration [14]. In a retrospective review of 382 patients undergoing retroperitoneal sarcoma resection, the authors found that this technique was associated with a 3-fold decrease in local recurrence in comparison to standard multi-organ resection only for tumor involvement. However, no difference in survival was noted. Santos et al. found that the technique of compartmental surgery did not appear to impact either recurrence rates or survival and was in fact, associated with higher intraoperative blood transfusion requirements and postoperative morbidity [15]. We did not utilize complete compartmental surgery in our series and believe that more studies are needed to validate its routine use.

Retroperitoneal tumors can also involve major abdominal vascular structures in up to 18% of patients [16], necessitating concomitant en bloc resection, as was done in five (8%) of our cases. Preoperative planning is critical and should include consultation with a vascular surgeon for resection and reconstruction. For right-sided tumors, the surgeon must be prepared to resect the inferior vena cava (IVC) when a solid mass abuts it. Most published reports in patients with non-hepatic, non-renal primary retroperitoneal tumors advocate use of synthetic graft (e.g. polytetrafluoroethylene, PTFE) to reconstruct the IVC [16-19]. Graft patency rates are very good (90-94%), as shown by studies with 19 to 36 months of follow-up review [16-19]. Alternatively, proximal ligation of the IVC after resection may be appropriate in select patients whose infrarenal tumors have extensive venous collateralization, or when concern for bowel anastomotic leakage would make synthetic graft placement risky. Minimal morbidity was reported in 11 patients who had proximal IVC ligation [20], but a subsequent report found that postoperative leg edema was twice as common after IVC ligation than after graft reconstruction [21]. Finally, although more rare and arguably more challenging, aortic resection for retroperitoneal tumors has also been described [16,22,23]. A variety of graft materials have been used for aortic resection, including Dacron [16], PTFE [16,22], polyethylene terephthalate [22], and even autologous superficial femoral vein [23]. Five patients with aortic resection and prosthetic reconstruction were reported to have a patency rate of 89% at 19 months [16]. For both venous and arterial reconstruction, the option of using cryopreserved human vein or extra-anatomic bypass (i.e. axillary-femoral) also exist, particularly when there is concern for enteric contamination within the resection site. In select cases, extracorporeal circulatory bypass may be helpful to permit complete and safe resection of retroperitoneal tumors with major vascular involvement [24].

In our series, complete resection was feasible for recurrent retroperitoneal tumors, although it was achieved slightly less often than for primary tumors (93% versus 97%, Table 1). Even more dramatic differences were reported by Lewis et al. more than a decade ago - 80% of patients with primary disease had complete resection versus 57% for those with local recurrence, and not surprisingly, the rate of complete resection continued to decrease with each subsequent recurrence [3]. Similar to our findings, more recent studies indicate a difference in the rate of complete resection, but suggest that the difference may not be as dramatic (99% versus 90%) [9]. The recent higher rates of complete resection for recurrent disease may reflect improved surgical technique, a more aggressive approach to resection of all retroperitoneal tumors, or improved patient selection. Complete resection should always be considered for recurrent disease as it is critical for improved survival [25] and in fact, may even result in comparable survival to patients after complete resection of primary disease [26].

Careful review of preoperative imaging is essential to anticipate potential operative scenarios and determine whether to utilize adjunct procedures to minimize complications. For example, ureteral stent placement and femoral nerve monitoring should be considered to identify these important structures when masses are near to their expected location in the retroperitoneum. Posterior laminotomy should be considered to permit complete resection of retroperitoneal tumors involving spinal nerve roots and when masses abut the vertebral bodies. Preoperative angioembolization of highly vascular tumors should be considered to minimize intraoperative blood loss.

Despite use of adjunct procedures, perioperative complications are common given the magnitude of the operations often required in retroperitoneal tumor resection. In terms of perioperative mortality, our 2% rate compares favorably to the 1-3% rates reported for large single institution patient cohorts [3,6] and a recent ACS-NSQIP national database review [27]. Our complication rate of 16% is also comparable to the recent 13-26% rates reported in the literature [6,27]. The caveat of reporting complications, of course, is that they are heavily dependent on the experience and expertise of the surgeon(s) and the resources of the institution, and are to some extent, driven by extent of the operation, which may differ from one patient to another.

Although not performed in our current series, laparoscopic resection of retroperitoneal tumors is emerging as a potential surgical option in select cases. Two recent series have suggested that in benign tumors without adjacent organ or vessel involvement, this approach can be done safely and result in good perioperative outcomes [28,29]. Tumor size does not appear to significantly affect blood loss or operative time, although the majority of tumors are relatively small (<10 cm). Laparoscopic resection of retroperitoneal liposarcoma [30,31] and leiomyosarcoma [32,33] have also been described in case reports. We feel that use of this approach in malignant disease should be tempered by the same oncologic principles of complete and safe resection and that there should be a low threshold for conversion to laparotomy in cases of actual or potential adjacent organ involvement.

## Conclusion

Our recent series of sixty-three cases of challenging primary and recurrent retroperitoneal tumor resections indicates that complete resection is feasible and can be done safely. The surgeon must take several factors into consideration, including the possibility of multi-organ and major vascular resection and the need for adjunct procedures to minimize complications. We fully advocate thorough preoperative planning and full utilization of institutional resources and consulting services on an individual patient basis.

## Competing interests

The authors declare that they have no competing interests.

## Authors' contributions

WWT participated in the design of the study, carried out the extraction and analysis of data, wrote the manuscript, and performed critical review of the literature. SCW, CME and RSW assisted in collection of the clinical data and assisted in reviewing the manuscript. EKN conceived of and participated in the design of the study, assisted in collection of clinical data, and assisted in reviewing the manuscript. All authors have read and approved the final manuscript.
